# High-dose pre-operative helical tomotherapy (54 Gy) for retroperitoneal liposarcoma

**DOI:** 10.1186/1748-717X-7-214

**Published:** 2012-12-17

**Authors:** Paul Sargos, Catherine Dejean, Bénédicte Henriques de Figueiredo, Véronique Brouste, Binh Nguyen Bui, Antoine Italiano, Eberhard Stoeckle, Guy Kantor

**Affiliations:** 1 Department of Radiation Oncology, Institut Bergonié, Bordeaux, France; 2 Université Bordeaux Segalen, 229 cours de l’Argonne, Bordeaux 33076, France; 3 Clinical and Epidemiological Research Unit, Institut Bergonié, Bordeaux, France; 4 Department of Medical Oncology, Institut Bergonié, Bordeaux, France; 5 Department of Surgery, Institut Bergonié, Bordeaux, France

**Keywords:** Retroperitoneal sarcoma, Liposarcoma, Pre-operative radiotherapy, Helical tomotherapy, Intensity modulated radiotherapy, Surgery

## Abstract

**Purpose:**

To evaluate the feasibility of pre-operative radiotherapy (54 Gy) with Helical Tomotherapy (HT) followed by surgery.

**Methods and materials:**

Ten patients with non-metastatic resectable retroperitoneal liposarcomas were treated by pre-operative tomotherapy (54 Gy) and surgery. Clinical and biological toxicities were evaluated on the CTCAEV3.0 scale. For nine patients, delivered tomotherapy plans were compared with retrospectively-planned dynamic intensity-modulated radiotherapy (IMRT) dosimetric studies.

**Results:**

No immediate or late Grade>2 toxicities were observed after radiotherapy. Post-operatively, one patient died and three patients experienced Grade 3 toxicity (two digestive and one metabolic). These toxicities disappeared and only two patients presented persistent Grade 1 paresthesia. R0 resection was obtained for four patients, R1 for four, and R2 resection for two. With a median follow-up of 26 months, no local or metastatic relapse was observed. Dosimetric comparisons between HT and retrospectively-planned IMRT demonstrate adequate target volume coverage for both techniques. Gastrointestinal sparing is higher with HT with a D200cc reduced by 5 Gy. Integral dose (ID) was increased in HT.

**Conclusions:**

High dose pre-operative radiotherapy (54 Gy) for retroperitoneal liposarcoma is feasible and mostly well tolerated. Cumulative toxicity and tolerance depend mainly on patient’s general status. Image-guided radiation therapy (IGRT) is essential, irrespective of the IMRT technique used. Furthermore, HT offers the possibility of sparing selected areas in such complex volumes.

## Background

Retroperitoneal sarcomas represent 12-15% of soft-tissue sarcomas and liposarcomas are the most common histological sub-type. They remain asymptomatic for an extended period and diagnosis is often made only when considerable growth becomes evident, involving or compressing the surrounding organs. With 5-year local recurrence rates varying from 35-85% according to different publications, local control is the principal aim for managing these tumours [1,2]. Treatment usually involves an en bloc surgery to remove the tumour and the adjacent viscera, however R0 resections are only achieved in 60-85% of cases [3-5]. To improve local control in retroperitoneal sarcomas, combination treatments including both surgery and radiotherapy have been developed (from strategies used for soft tissue sarcoma of the extremities).

One of the major obstacles for radiotherapy (pre-, intra- or post-operative) is the proximity of adjacent organs. Reports from studies delivering 45 to 50 Gy have not confirmed the efficacy of radiotherapy in the treatment of these tumours, nor defined the best therapeutic sequence [6,7].

In this study we examined high-dose pre-operative irradiation of retroperitoneal liposarcoma. The principal objective was to evaluate the feasibility of high-dose pre-operative irradiation by tomotherapy at 54 Gy by analysing immediate and late clinical tolerance after radiotherapy and after surgery. Secondary objectives were to evaluate the quality of the surgery, local control and overall survival (OS) rates. Finally, dosimetric comparisons were made between the helical plan by HT used for treatment with retrospectively-planned conformational sliding window intensity-modulated radiotherapy (IMRT).

## Methods and materials

Ten patients with newly diagnosed retroperitoneal liposarcoma treated consecutively with pre-operative radiotherapy (HT) and surgery at our Institution between August 2007 and September 2008 were included in this pilot, prospective study. All patients were treated by a unique team consisting of a radiation oncologist and a surgeon. No chemotherapy was delivered. Patients were receiving first-line treatment, did not have any metastases, and presented with tumours that the surgeon judged to be resectable. Three patients with multiple focality comprising of sarcomatosis, or history of complicated prior laparotomies with extensive adhesions precluding extended field radiotherapy, were excluded. The anatomopathologic characteristics are consistent with the French National Comprehensive Cancer Care Centre (FFCLCC) classification [8]. Patient characteristics are described in Table 1. Institutional review board approval was obtained for this pilot study.

**Table 1:** Patients and Tumour characteristics for retroperitoneal liposarcoma patients treated with pre-operative helical tomotherapy

**Patient**	**Gender**	**Age (yrs)**	ECOG*	ASA**	**Tumour size (cm)**	**Side**	**Histology**	**Grade**
1	F	39	1	1	20	Right	Dedifferentiated liposarcoma	3
2	F	62	1	1	26	Left	Dedifferentiated liposarcoma	3
3	M	69	1	1	11	Left	Well-differentiated liposarcoma	1
4	M	48	1	2	29	Right	Dedifferentiated liposarcoma	3
5	M	73	1	2	19.5	Left	Dedifferentiated liposarcoma	3
6	F	78	1	2	30	Left	Dedifferentiated liposarcoma	3
7	F	51	1	1	25	Left	Well differentiated liposarcoma	1
8	F	50	1	1	22	Right	Dedifferentiated liposarcoma	1
9	M	79	1	2	30	Right	Dedifferentiated liposarcoma	3
10	F	52	1	1	40	Right and left	Well-differentiated liposarcoma	1

*Eastern Cooperative Oncology group.

**American Society of Anesthesiologists.

### Treatment

#### Pre-operative radiotherapy by HT

Computed tomography (CT) images were acquired in two series, before and after intravenous injection to identify the vascular axis. Contours of the clinical target volume (CTV) were defined jointly by the surgeon and the radiation oncologist. The CTV corresponded to the gross tumoral volume (GTV) represented by a high density zone on the CT scan related to a dedifferentiated component. This CTV also encompasses the adjacent fatty structure assumed to be involved. Contact areas such as posterior wall, adjacent viscera (especially kidney, large bowel and small bowel) were included, as were the zones of contact between the lesion and the directly adjacent organs (zones at high risk of R1 resection). Limits between normal fat and pathologic fat were occasionally difficult to define. The planning target volume (PTV) was obtained by a 5mm 3D geometric expansion from the CTV (except for one patient). This margin was determined after observation of interfraction tumor motion of retroperitoneal tumour patients treated by tomotherapy in the department. This small margin is justified by the limited mobility of these tumours that are restricted within a confined space as well as by the daily position monitoring in tomotherapy. The specific organs at risk (OAR) and constraints are described Table 2. Small bowel loops were excluded from the PTV and a virtual space corresponding to the small bowel without PTV was created.

**Table 2:** Dosimetric constraints utilized for tomotherapy planning for pre-operative helical tomotherapy

	**Index**	**Expected value**	**Definition**
**Target volume**			
-Planning Target Volume (PTV)	V95	>95%	Volume receiving at least 95% of prescribed dose
	D95	>95% (51.3 Gy)	Dose received by 95% of the volume
	D98	>90% (48.6 Gy)	Dose received by 98% of the volume
	D2	<107% (57.8 Gy)	Dose received by 2% of the volume corresponding to the “hot spots”
	D_mean	54 Gy	Average dose (close as possible to the prescribed dose)
**Organs at risk**			
-Gastrointestinal [9]	D200cc	<50 Gy	Dose received by 200cc of the intestinal volume
	D2	<54 Gy	Dose received by 2% of the volume corresponding to the “hot spots”
	V45	<33%	Volume receiving 45 Gy
	V20	<50%	Volume receiving 20 Gy
-Contralateral kidney	D2	<12 Gy	Dose received by 2% of the volume corresponding to the “hot spots”
-Spinal canal	D2	<45 Gy	Dose received by 2% of the volume corresponding to the “hot spots”

Treatment planning was carried out with the Tomotherapy HiArt dedicated inverse planning system. For each patient this planning was undertaken using a pitch of 0.3 and a field width of 2.5cm, except for one patient where a field of 5cm was necessary to minimise treatment duration. On average, the modulation factor was 1.62. The dose, prescribed at the median PTV (as recommended for the ICRU report n°83 dedicated to IMRT), was 54 Gy, delivered in 30 fractions of 1.8Gy, five days weekly. The review of the dose volume histograms and of different dosimetric indices (Table 2) also enabled a quantitative evaluation of treatment plans [10].

Before each irradiation session, a MegaVoltage scan was carried out for each patient with the compact HT accelerator (using 3.5 MV energy). A realignment of this scanner, based firstly on an analysis of the concordance of bone structures, but also on the contralateral kidney and the target volumes, was carried out.

### Surgery

Each patient was seen twice by the surgeon, once before any treatment, to determine whether the tumour was resectable, and then after radiotherapy to confirm the indication for surgery. Surgery was performed on average 4 weeks after radiotherapy (from 3 to 6 weeks after). It consisted of a midline incision for patients in the supine position then an en bloc resection of the tumour and involved adjacent organs. Only involved adjacent viscera were removed, but systematic compartmental resection was not the goal. Quality of the resection was defined according to the UICC R (residual tumour) classification (R0: in sano, R1: microscopic residue, R2: macroscopic residue).

### Methodological considerations

The principal objective of this study was to evaluate the feasibility of pre-operative HT treatment at 54 Gy. Tolerance of the therapeutic sequence was evaluated weekly during radiotherapy, after completion of the radiation treatment, and after surgery using the NCI’s CTCAE v3.0 scale. Late tolerance was evaluated alternately by the surgeon and the radiotherapist every three months for the first year and then every six months. Patient follow-up consisted in a full clinical examination and regular blood tests with haematology, electrolytes, kidney function, hepatic function and serum amylase. Secondary objectives were to evaluate surgery quality, recurrence-free (RFS) and overall (OS) survivals. Tomotherapy plans were also compared with retrospectively-planned dynamic IMRT treatment to define the optimal radiation technique.

#### Dosimetric comparison between tomotherapy and dynamic IMRT

Comparative Dynamic IMRT planning was performed for all patients using the VARIAN® Eclipse-Helios software with 5 equally distributed fields of 18MV X-photons. The dosimetric constraints used were the same for tomotherapy planning. For one patient, it was not possible to perform the IMRT dosimetry due to the mechanical characteristics of the multi-leaf collimator smaller than the size of the largest tumoral axis. Nine patients were therefore included for these analyses. The different indices used for this comparison are displayed in Table 3 as defined in a previous publication [11]. The minimum PTV dose was defined as the D98%. The contralateral kidney was evaluated with the Equivalent Uniform Dose (EUD). Dosimetric data were compared using the non parametric Wilcoxon’s test at the p<0.05 significance level.

**Table 3:** Results of the dosimetric comparison between tomotherapy and intensity-modulated radiotherapy (IMRT)

** Volume**	** Indices**	**Tomotherapy**	**IMRT**
Planning Target Volume (PTV)	V95 (%)	97	96.3
	D95 (Gy)	52.4	51.7
	D98(Gy)	50.6	50.9
	D2 (Gy)	55.2	54.6
	D_mean (Gy)	53.8	53.4
	SD*	1.06	0.92
	DSC†	0.88	0.91
	HI††	0.086	0.068
Gastrointestinal	D2 (Gy)	50.8	51.8
	V45 (%)	**7.3**	**10.3**
	V20 (%)	45.6	45.3
	D200cc (Gy)	**41.3**	**46.5**
Contralateral kidney	D2 (Gy)	8.3	8.9
	EUD§ (Gy)	3.9	3.5
Spinal canal	D2	**32.5**	**39.1**
Healthy tissue	Integral dose (Joules)	**354.1**	**294.7**
	Integral dose RVRII (Joules)	**321.5**	**259.6**

* SD=Standard Deviation, †DSC=Dice Similarity Coefficient, †† HI=Homogeneity Index, § EUD= Equivalent Uniform Dose, II RVR= Remaining Volume at Risk.

(Results in bold indicate statistical significance at the p<0.05 level).

## Results

### Feasibility and tolerance of the HT treatment

#### Tolerance during and after radiotherapy

Radiotherapy was well tolerated. All patients finished their treatment without interruption and no Grade >2 toxicity was observed. Grade 1 fatigue and asthenia were described by all patients. The main toxicities observed were digestive. Seven patients showed Grade 1 toxicity such as anorexia, nausea, vomiting or diarrhoea. Symptoms were managed with anti-emetics (metoclopramide, setrons). One patient presented with Grade 2 nausea and anorexia. At the end of the radiation, the average weight loss was 2.5 kg (range: 0–8 kg). One patient presented with cutaneous toxicity (lower abdomen) in the form of Grade 1 erythema and one patient showed transitory Grade 1 toxicity corresponding to the beginning of renal failure, probably of a functional origin linked to hypovolemia caused by the diarrhoea, vomiting, and weight loss. No changes were observed in pancreatic or liver function tests.

### Results and immediate tolerance of surgery

R0 surgery was obtained for four patients, R1 for four patients and R2 for two patients. Resection of the adjacent organs was required due to tumoral size, localisation and local involvement for the: ipsilateral kidney (10), adrenal gland (7), hemi-colon (3), adnexa (2), and spleen (2). Muscle resection concerned mainly the psoas muscles.

Mean hospital stay was eight days. One patient died 36 days after surgery, from a cerebral haemorrhage due to thrombopenia in the context of sepsis and a disseminated intravascular coagulation syndrome. The patient was 73-years-old, had a WHO score of 1 and ASA score of 2, with a tumour in close contact with the iliac vessels. Two other patients had Grade 3 digestive toxicity with significant weight loss. Five patients displayed Grade 1 digestive toxicity. Mean weight loss was 2 kg after surgery (but the weight of the surgical specimen removed must be taken into account). Two patients experienced pain and Grade 1 neuropathies in a form of dermatomal paresthesia after resection of nerves in the surgical area. This symptomatology was a consequence of tumour involvement of the psoas muscle that was manipulated during surgery. Finally, one patient presented with a Grade 3 renal failure (creatinine clearance = 28ml/min). This patient was 77-years-old and had received surgery that was macroscopically complete but had a total unilateral nephrectomy. This renal failure was probably linked to a multifactor kidney malfunction (age, hypovolemia and nephrectomy).

### Tumour control and late toxicity

No patient was lost to follow-up. With a median follow-up of 26 months (range: 12–36), no local or systematic recurrence was observed and all nine surviving patients were in a good general state. No R2 patients presented local relapses. In terms of delayed toxicities, only two patients presented persistent and non invalidating pain and paresthesia in manipulated and irradiated zones. The other toxicities described during the immediate postoperative period were all resolved. At the moment of the last evaluation, patients had gained an average of three kilos.

### Comparative dosimetric analysis

Table 3 summarises the tomotherapy and IMRT dosimetric data for each patient. All dosimetric constraints were respected for the two techniques. For the PTV, the V95%, D95%, D2% and D_mean are not significantly different across techniques. The minimum PTV dose was always superior to 95% of the prescribed dose. Prescription homogeneity evaluated through the Homogeneity Index (HI) is the same for the two techniques. In terms of conformity, the IMRT seems to offer better results given the higher Dice Similarity Coefficient (DSC) evaluated on the 95% isodose (0.91 compared to 0.88 in tomotherapy, p=0.0284).

**Figure 1:**
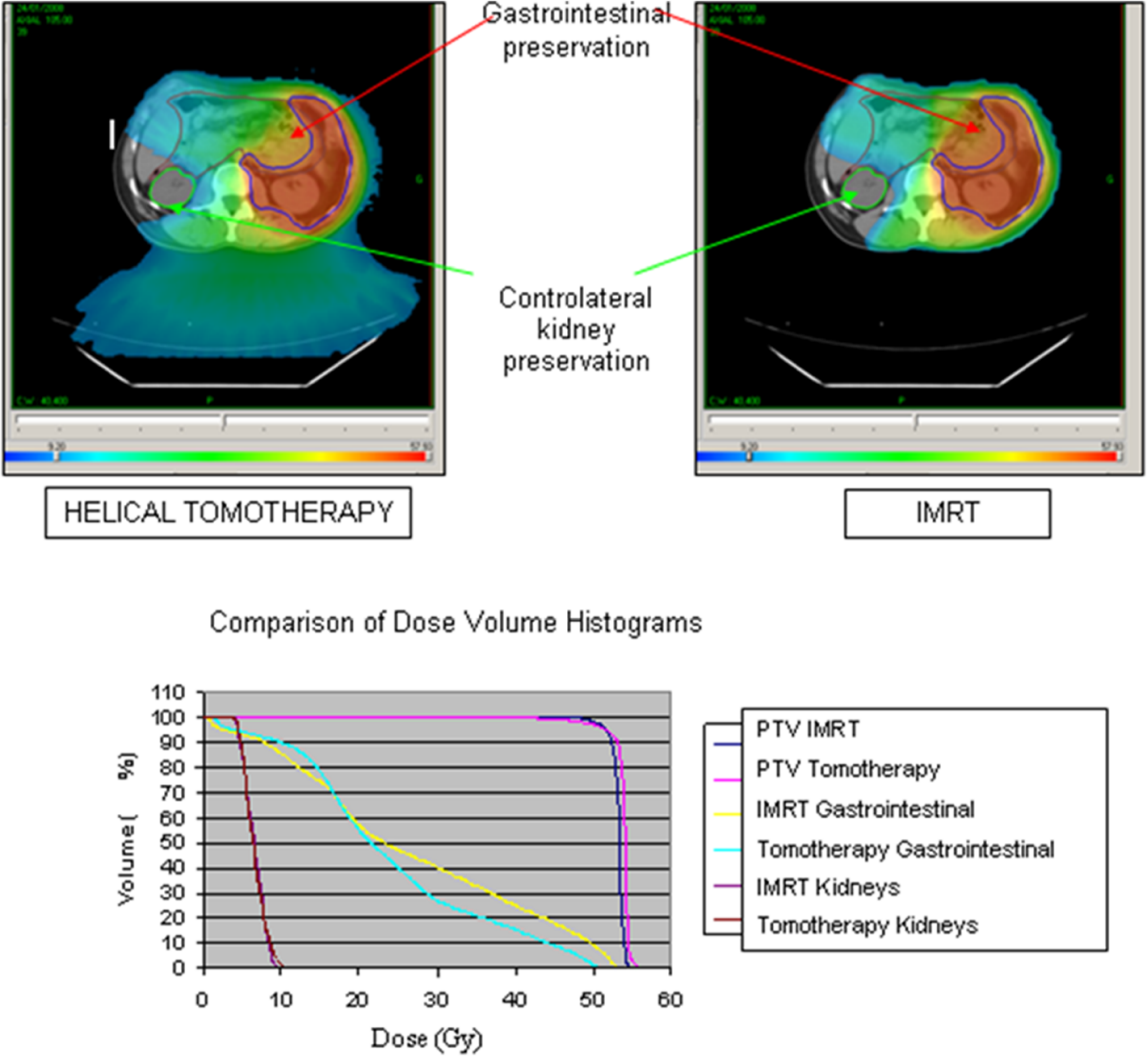
Dosimetric comparison between Tomotherapy and intensity-modulated radiotherapy (IMRT) for a left retroperitoneal liposarcoma (for the same prescribed dose of 54 Gy, note the greater gastrointestinal preservation with helical tomotherapy and the equivalent controlateral kidney preservation).

With a V45Gy of 10.3% in IMRT and 7.3% in tomotherapy (p=0.05), and a D200cc lower by 5Gy in tomotherapy (p=0.021), gastrointestinal sparing was better in tomotherapy. The maximum dose received by the spinal canal was reduced in tomotherapy (39.1Gy in IMRT compared to 32.5Gy in tomotherapy; p=0.038), even though both of these values are far from toxicity values (Dmax<45Gy). Finally, there were no significant differences between the two techniques concerning sparing of the contralateral kidney (evaluated by the D2% and the EUD) (Figure 1).

In terms of the integral dose (ID) administered to healthy tissue, the comparative analysis is in favour of IMRT regardless of the calculation method utilised (D’souza et al. [12] or ICRU N°83). Dynamic IMRT with equally-distributed fields of 18MV photons enables the reduction of the ID to healthy tissues by 60 joules on average (p=0.008) compared to HT (Figure 1).

## Discussion

The mainstay of treatment of retroperitoneal liposarcomas is surgery [13,14]. Due to rarity of these tumours, data on radiotherapy are limited and many questions remain unanswered. In the current series focussing on retroperitoneal liposarcoma treated by high-dose **pre-operative Helical Tomotherapy**, toxicity was low to moderate and mostly grade<3 on the CTCAEV3.0 scale. During radiotherapy and after surgery, toxicities (which were mainly digestive), were managed by symptomatic treatments. After radiotherapy, no Grade>2 toxicity was observed nor any delayed digestive toxicity. No occlusion or perforation occurred. Furthermore, R0 surgery was achieved in 40% of cases, R1 for 40% and R2 in the remaining 20%. With a median follow-up of 26 months, no local recurrence occurred.

### Radiotherapy schedule

No randomised study has yet compared results from radiotherapy administered at different stages of treatment (pre-, intra- or post-operative) [15]. Further, recurrence tends to occur at distance after pre-operative radiation, whereas it is more likely to be local after post-operative radiation [16]. Pre-operative radiation appears to offer several advantages compared to post-operative radiation. The OAR (intestines, liver), repressed by the tumour are more easily preserved and toxicities, particularly digestive, are reduced. Bossi et al.’s series evaluating pre-operative voluntarily partial radiation at 50 Gy shows good tolerance because less than 10% of patients presented with Grade 3 digestive toxicity [17]. In contrast, post-operative radiation seems to result in greater digestive toxicity. In a series of 45 patients receiving post-operative radiation of 40.8 to 59.4 Gy, Gilbeau reports 42% of Grade 2 digestive toxicity [18].

### Dose level

Results concerning local control in our series may indicate the superiority of a high dose (54 Gy), but this would need to be confirmed in a larger prospective trial. Data from the French multicentre TOMOREP protocol are expected shortly. Local control appears dose-dependent [19]. For 104 patients receiving radiation after complete surgery, Catton et al. showed that time to local recurrence was 30 months after low dose radiation (under 35 Gy) vs. 103 months after radiation at doses higher than 35 Gy (p=0.06) [1].

The increase in pre-operative or post-operative radiation doses requires the use of new radiation techniques (intensity modulation) to allow sufficient sparing of healthy surrounding tissues. Theoretical and clinical dosimetric studies have already shown that the doses delivered can be increased with IMRT. In a previous study [6], the post-operative use of IMRT enabled the dose to be raised to 54 Gy instead of 45 Gy in 3-dimensional conformational radiotherapy (3D-CRT) while improving the protection factor for surrounding organs by 20%. The dose reductions outside the PTV enabled this prescribed dose increase of 9 Gy to reach 54 Gy [6]. Similar results were found by Koshy et al. [20] or in Bossi et al.’s series with increased gastrointestinal and renal sparing on dosimetric comparisons between 3D-CRT and IMRT for 10 patients (dose of 50.4 Gy). With the same dosimetric limitations used for 3D-CRT planning and in IMRT, the average dose to the small intestine decreased from 36 Gy in 3D-CRT to 27 Gy in IMRT. Tumoral coverage (V95) is improved with IMRT, increasing from 95.3% to 98.6%. Pre-operatively, Bossi et al. also found greater renal sparing with IMRT (kidney volume receiving more the 15 Gy reduced from 19.8cc in RTC3D to 3.2 cc with IMRT) [17].

Similarly, Tzeng et al. report the results of 16 patients receiving radiation at 57.5 Gy. Local control was 80% with only 25% of patients experiencing Grade 2 digestive toxicity probably due to the fact that the high-dose PTV was smaller than the standard PTV, and intentionally far from the small bowel [7].

### IMRT techniques

Technological improvements in radiotherapy are ever-increasing and intensity modulated treatment can be administered by various means (tomotherapy, dynamic IMRT, arc therapy, etc.) [21]. The comparative dosimetry study shows very good tumoral coverage for the two techniques examined, with all dosimetric evaluation constraints defined by the ICRU N°83 respected. In particular, homogeneity of coverage, evaluated through HI, is similar across IMRT and tomotherapy. In terms of conformity, the DSC (calculated with the 95% isodose) is better after IMRT. The use of the 95% isodose for this comparison is, however, questionable given the possibilities of optimisation offered by these modern techniques. When evaluation isodoses are increased progressively from 95 to 100% to calculate the DSC, thus increasing the constraints and conformational quality, the conformity index approaches the ideal value of 1 in tomotherapy, whereas in IMRT it moves away from 1 up until the isodose of 98% (p=0.051). In terms of OAR-sparing, digestive organs are spared more in tomotherapy (D200cc lowered by 5 Gy). Given the short duration of follow-up, it is however impossible to know the clinical consequences of this dosimetric gain.

It is also particularly important to evaluate the “low doses” delivered during the intensity modulation treatment [22]. Using a biological model, Hall et al. estimate that the risk of a second cancer at 10 years is potentially doubled when an IMRT technique is used compared to a 3D-CRT across all pathology types [23]. Our comparison indicates that the integral dose delivered is weaker (on average 60 Joules) in dynamic IMRT than in tomotherapy, irrespective of the calculation method used (D’Souza or Remaining Volume at Risk, RVR). The integral dose seems to increase with tomotherapy when similar energy is used. The larger field length (in a longitudinal direction) of the tomotherapy plans relative to retrospectively-planned IMRT could be one reason for these higher integral dose values [22].

However, intensity modulation treatments, given the high gradients, must be accompanied by daily position monitoring to ensure accurate target positioning. In tomotherapy this is ensured by daily megavoltage CT scans and during arc therapy by daily cone beam CT scans (CBCT) [24]. Overall, the evaluation of the integral dose delivered to healthy tissues by a treatment must take into account both the energy emitted by the radiation itself but also that emitted by the repositioning imagery [25].

## Conclusions

The low toxicities in our series, both during and immediately after radiotherapy and after surgery, show the technical and clinical feasibility of a pre-operative radiation strategy at a high dose of 54 Gy for patients with retroperitoneal liposarcoma. Clinical evaluation of the age and general status of patients is essential to judge feasibility. This irradiation must be delivered with intensity modulation to allow for sufficient sparing of surrounding at-risk organs. Tomotherapy, including image-guided radiation therapy (IGRT) systems, appears to be an attractive option with regard to gastrointestinal preservation, especially in large and convex volumes. This technique enables us to treat safely a larger number of patients who would not otherwise have been able to receive radiation. With regard to the delayed effects of integral dose, only long-term and prospective data will answer this specific question. In terms of the cancer outcomes in this series, no local or distant recurrence was observed with a median follow-up of 26 months, but longer follow-up needs to be obtained to confirm these preliminary results.

## Abbreviations

CTCAE: Common terminology criteria for adverse events; ICRU: International Commission on Radiation units; IMRT: Intensity-modulated radiotherapy; MV: Mega volt; ID: Integral dose; HT: Helical Tomotherapy; Gy: Grays; IGRT: Image-guided radiation therapy; OS: Overall survival; CT: Computed tomotherapy; RVR: Remaining risk volume; DSC: Dice similarity coefficient; 3D -CRT: Three dimensional conformational radiotherapy; PTV: Planned target volume; OAR: Organs at risk; GTV: Growth tumoral volume; CTV: Clinical target volume; NCI: National Cancer Institute; EUD: Equivalent Uniform Dose; WHO: World Health Organisation; ASA: American Society of Anesthesiologists; HI: Homogeneity Index.

## Competing interests

The author’s declare that they have no competing interests.

## Authors’ contributions

PS conceived of the study, participated in its design and coordination and helped to draft the manuscript. CD supervised the technical and theoretical technique of the physical data. BHdF participated in the design of the study and helped to review the manuscript. VB participated in the design of the study and performed the statistical analysis. BNB, AI, ES and GK conceived of the study, participated in its design and coordination and helped to draft the manuscript. All authors read and approved the final manuscript.
